# Assessment of occupational stress among certified registered anesthetists in the Greater Accra region

**DOI:** 10.3389/fpubh.2024.1335948

**Published:** 2024-02-14

**Authors:** Dorcas Edem Sabblah, Abdulai Mohammed Salifu, Richmond Owusu, Serwaa Akoto Bawua

**Affiliations:** ^1^Department of Biological, Environmental and Occupational Health Sciences, School of Public Health, University of Ghana, Legon, Accra, Ghana; ^2^General Surgery Unit, Surgical Division, 37 Military Hospital, Negheli Barracks, Accra, Ghana; ^3^Department of Health Policy, Planning and Management, School of Public Health, University of Ghana, Legon, Accra, Ghana

**Keywords:** occupational stress, certified registered anesthetists, anesthesia practice, work related stress, health facility, standard equipment, health, work performance

## Abstract

**Background:**

Work-related stress is a prevailing concern within the community of Certified Registered Anesthetists (CRAs), significantly impacting both the health and professional performance of these individuals. This study aimed to assess work-related stress and its influencing factors among CRAs practicing in the Greater Accra region was examined.

**Methods:**

Using convenience sampling techniques, data were gathered from 140 participants via a Google form questionnaire distributed through WhatsApp. Descriptive statistics were employed to analyze the collected data, focusing on frequencies and proportions for categorical variables. For continuous variables, bivariate analysis (Chi-square) and ordinal logistic regression were conducted using STATA 16. A *p*-value <0.05 was considered significant.

**Results:**

Among the 140 CRAs, 20 individuals (14.3%) reported experiencing mild stress levels according to the Weiman Occupational Stress Scale. Approximately 3 out of 4 CRAs (73.6%) reported having moderate stress levels, and 12.1% reported severe stress levels. This indicated that the majority of CRAs experienced moderate levels of stress, which was notably affected by the type of health facility and the use of inadequate or sub-standard equipment in the hospitals.

**Conclusion:**

Based on these findings, the study recommends educational programs and counseling for CRAs to heighten awareness of the demanding nature of their job. Additionally, it suggests the provision of proper resources and standard equipment for CRAs. Facility-level motivation for CRAs is also advised to alleviate their stress. Finally, the study proposes further investigations into the factors contributing to work-related stress among CRAs.

## Introduction

Work-related stress among Certified Registered Anesthetists (CRAs) is a prevalent issue contributing to health problems and a decline in work performance. Internationally recognized as one of the most stress-inducing professions, anesthetist work significantly impacts the physical and psychological well-being of practitioners (1). The significance of work-related stress is paramount for employers, affecting employee effectiveness and job satisfaction, often resulting in decreased work performance (2). Stress, the interaction between individuals and their physical environment, can significantly impact lives, leading to adverse health outcomes. Stressors, triggering physiological reactions like increased heart rate, elevated blood pressure, and heightened pulse rate, also elicit psychological responses such as anxiety, frustration, and anger in affected individuals (3).

While work is a necessity for livelihood, the stress experienced during work should not jeopardize individual health. Stress significantly impacts an individual’s well-being, especially their cardiovascular health, as the heart is subjected to numerous stressors that can lead to cardiovascular diseases. This is evident in stress-induced symptoms like anxiety, sleep disturbances, and excessive rumination, which directly affect heart health (4). The stress experienced by Certified Registered Anesthetists (CRAs) is a result of the psychological and physical events encountered during their work and their environmental interactions, particularly within the hospital setting, where workloads often exceed manageable levels (5).

Research by Govender et al. (6) highlights the prevalence of stress and burnout among healthcare professionals, with doctors and nurses facing notably high-stress levels. While the general labor force’s estimated work-related stress levels are around 18%, doctors have a much higher rate, at approximately 28% (6). Specifically, 51% of doctors were found to be stressed, with 27% experiencing extreme stress (morbid stress) in a study comprising 67 doctors (6). Stress in doctors can lead to various negative consequences, underlining the importance of early stress detection for the well-being of doctors, their families, and their patients. Though there is existing data on work-related stress among doctors and nurses in Ghana, limited information is available concerning Certified Registered Anesthetists. Therefore, there is a need to gather more comprehensive data on work-related stress among CRAs. The objective of this study is to identify the factors associated with work-related stress among CRAs in the Greater Accra region.

## Methods

### Study setting

**Figure 1:**
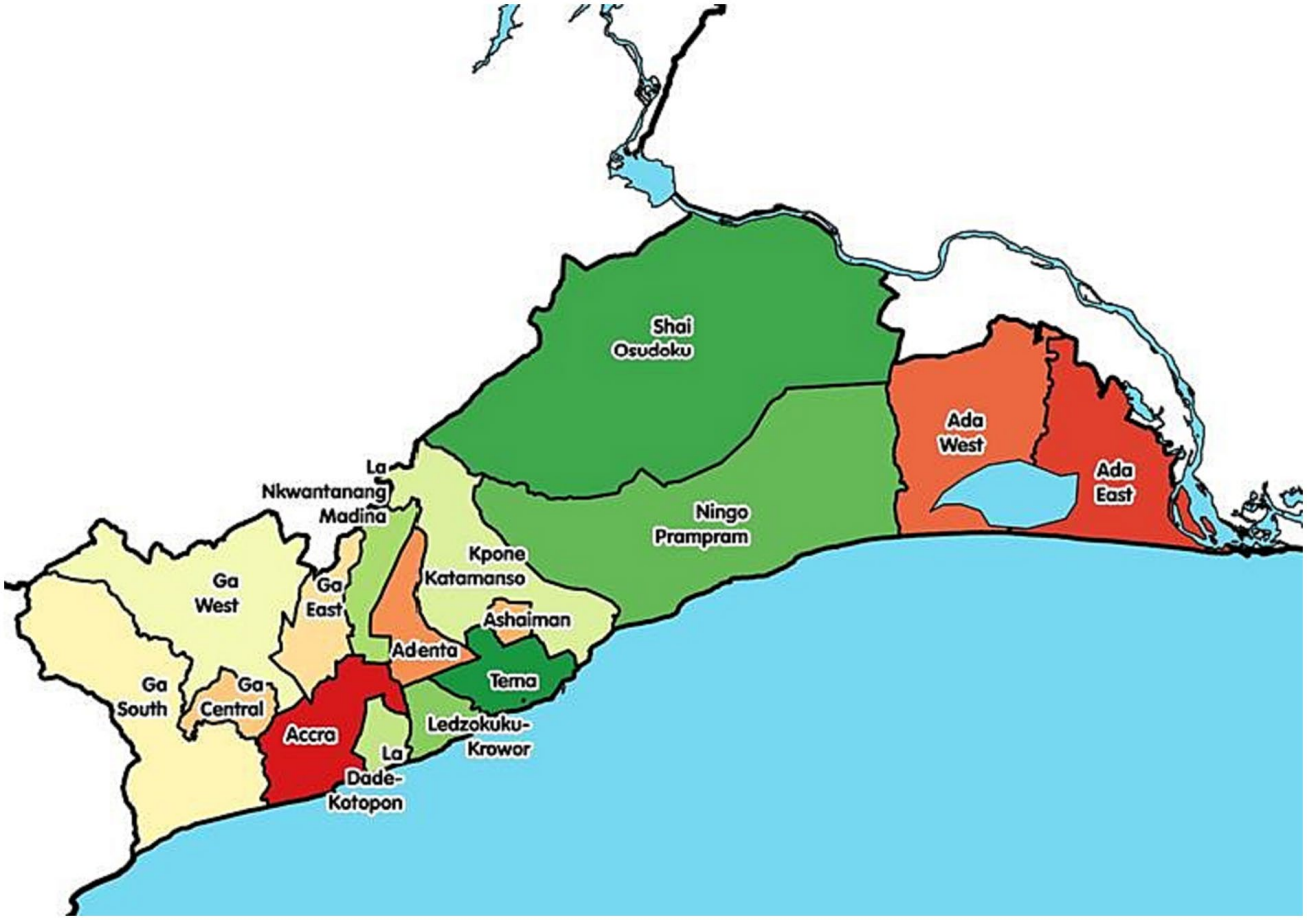
Map of Greater Accra region, which is the study area.

Greater Accra is one of the most populous cities in Ghana. Greater Accra consists of 19 districts with 73 district healthcare facilities. These healthcare facilities include clinics, hospitals, Community-Based Health Planning Services (CHPS), polyclinics, maternity homes, private medical centers, and the Christian Health Association of Ghana hospitals. Aside from these healthcare facilities, Greater Accra has one regional hospital (1), one teaching hospital (1) and one military hospital (1). The CRAs in the Greater Accra region were chosen because they serve a large population, the workload is strenuous, and the CRAs are overburdened (Figure 1).

### Study design and population

This study used a quantitative cross-sectional study design to assess work-related stress among CRAs practicing in the Greater Accra Region licensed by the Ghana Medical and Dental Council (GMDC) (Medical and Dental Council). The study population was taken from the 2021 gazette of Ghana Medical and Dental Council (GMDC), where a total of 1,244 CRAs were permanently registered in Ghana, and 198 CRAs were said to be in Greater Accra Region as of 2021 (7).

### Sample size

This study used a census in recruiting participants due to the relatively small size of the study population and also to ensure a comprehensive representation and minimize sampling error. There were 198 certified registered anesthetists as of 2021 in the Greater Accra region. Out of the 198 CRAs, 140 CRAs volunteered to participate in the study.

### Data collection and sampling process

A sample of 140 Certified Registered Anesthetists (CRAs) was selected from a total of 198 CRAs in the Greater Accra region using a convenience sampling technique. The study was conducted over the period between June and December 2021 in the Greater Accra region of Ghana. Participants included anesthetists from various healthcare settings such as regional, teaching, military, district, CHAG, Quasi, and private hospitals in the region. The questionnaire was disseminated electronically through WhatsApp and e-mail for all respondents who volunteered to participate. Employing WhatsApp and e-mail facilitated seamless and user-friendly communication, enabling participants to conveniently access and respond to the questionnaire at their own convenience, particularly during the challenging circumstances posed by the COVID-19 pandemic during the period of the study. Moreover, the use of these electronic platforms ensured that the researchers could address any queries and encourage timely responses, enhancing the efficiency of data collection.

The structure of the questionnaires was derived and adapted from previous studies, particularly those developed by Bakshi et al. (8). The questionnaire included three distinct sections: a socio-demographic profile, work-related stress evaluation, and identification of factors associated with work-related stress among Certified Registered Anesthetists (CRAs). The first section gathered socio-demographic information such as age, gender, marital status, income level, professional rank, years of experience, number of dependents, part-time job involvement, hours worked per shift, and the type of health facility where the participant was employed. The second part of the questionnaire aimed to assess the level of work-related stress experienced by CRAs. It consisted of Likert-scale questions designed to measure stress levels among CRAs. The Likert scale used was constructed based on the Weiman Occupational Stress Scale. It served as a tool to gage the stress levels experienced by CRAs.

The work-related stress section comprised nine Likert-scale questions, with participants rating their stress levels on a scale of 1 to 5. The ratings corresponded to 1 = never, 2 = rarely, 3 = occasionally, 4 = frequently, and 5 = always. The third section focused on identifying specific factors associated with work-related stress. This segment included 10 questions, with participants responding in the format of ‘yes’ or ‘no’ regarding factors contributing to work-related stress among CRAs.

### Data analysis

The collected data were initially categorized and coded numerically using Microsoft Excel spreadsheet window 10. Subsequently, the data was imported into STATA version 16 for analysis. Descriptive analysis was performed, and the outcomes were presented in mean, standard deviation, frequencies, and percentages.

To explore the relationship between factors associated with work-related stress and stress levels, a Chi-square test was conducted. A *p*-value of less than 0.05 was considered statistically significant. Variables such as type of health facility, inadequate or sub-standard resources, equipment, and supplies, and working alone on difficult cases displayed statistical significance in the Chi-square test. They were further examined using an ordinal logistic regression model. This model was designed with stress level as the dependent variable and all other variables as independent variables, aiming to determine if they remained statistically significant. The results were conveyed with a 95% confidence interval and reported odds ratio (OR).

### Ethical statement

The study obtained ethical clearance from the Ghana Health Services Ethics Review Committee, with certificate number GHS-ERC: 056/09/21. Also, written informed consent was obtained from all participants. Participants were informed that participation was voluntary and that they could opt-out at any time. Data collected was kept confidential, and participants were identified using codes and numbers instead of their actual names to ensure anonymity.

## Results

### Socio-demographic characteristics of CRAs

Out of 140 respondents, 54.3% were female and 45.7% were male. The majority were between 31 and 40 years old (54.3%), followed by over 40 years (26.4%) and 20–30 years (19.3%). Regarding marital status, 19.3% were married, 22.9% were divorced/separated, and 57.9% were single/cohabiting. In terms of rank, 50.7% were Principal CRAs, 25.7% were Senior CRAs, 14.3% were Certified Registered Anesthetists, and 9.3% were Deputy Chief CRAs. Work experience varied, with 36.4% having 0–3 years, 33.6% with 4–7 years, 16.4% with over 10 years, and 13.6% with 7–10 years. Income correlated with rank, with 47.1% of Senior CRAs earning 2,501–3,500 GHC, 25% of Principal CRAs earning 3,501–4,500 GHC, 16.4% of Certified Registered Anesthetists earning 1,500–2,500 GHC, and 11.4% of Deputy Chief CRAs earning more than 4,501 GHC. Work settings included district hospitals (35%), teaching hospitals (17.9%), 37 Military hospitals (17.1%), Greater Accra Regional hospitals (22.9%), and quasi/private/polyclinics (7.1%). The majority (62.9%) worked between 41 and 50 h per week, and the number of locums undertaken showed that 47.9% had 0–1 locums, 42.1% had 2–3 locums, and 10% had more than 4 locums. Additionally, 55.7% had 3–4 dependents, 34.3% had 0–2 dependents, and 10% had more than 4 dependents, influencing their need for additional income (Table 1).

**Table 1:** Socio-demographic characteristics of CRAs.

Characteristics	Frequency	Percentage
	(*N* = 140)	
Sex
Female	76	54.3
Male	64	45.7
Age
20–30	27	19.3
31–40	76	54.3
>41	37	26.4
Marital status
Single/Cohabiting	81	57.9
Married	27	19.3
Divorced/Separated	32	22.9
Number of dependents
0–2	48	34.3
3–4	78	55.7
>4	14	10.0
Income level (GHS)
1,500–2,500	23	16.4
2,501–3,500	66	47.1
3,501–4,500	35	25.0
>4,500	16	11.4
Rank/Grade
CRAs	20	14.3
SCRAs	36	25.7
PCRAs	71	50.7
DCCRAs	13	9.3
Type of health facility
District hospital	49	35.0
37 Military hospital	24	17.1
Regional hospital	32	22.9
Teaching hospital	25	17.9
Quasi/Private/Polyclinic	10	7.1
Years of experience (Years)
0–3	51	36.4
4–7	47	33.6
7–10	19	13.6
>10	23	16.4
Average number of hours spend at work (hours)
<40	8	5.7
41–50	88	62.9
51–60	33	23.6
> 61	11	7.9
Number of part time jobs
0–1	67	47.9
2–3	59	42.1
>4	14	10.0

### Work-related stress levels

Among the 140 Certified Registered Anesthetists (CRAs), 20 individuals (14.3%) reported experiencing mild stress levels according to the Weiman Occupational Stress Scale. Approximately 3 out of 4 CRAs (73.6%) reported having moderate stress levels, and 12.1% reported severe stress levels. This demonstrates that the prevalent stress level among CRAs is primarily moderate. Breaking down the stress levels by sex, of the 76 female CRAs, 13 individuals (17.1%) reported mild stress, the majority of 54 CRAs (71.1%) reported moderate stress, and 9 individuals (11.8%) reported severe stress. Similarly, the data indicates that the majority of female CRAs experienced moderate stress levels. Moreover, out of the 64 male CRAs, 7 individuals (10.9%) reported mild stress levels, while 3 out of every 4 males (76.6%) reported moderate stress levels, and 12.5% reported severe stress levels. Once again, the data indicates that the majority of male CRAs reported experiencing moderate stress levels (Table 2a).

**Table 2a:** Work-related stress level among CRAs.

	Total	Mild	Moderate	Severe
	140	20 (14.3%)	103 (73.6%)	17 (12.1%)
Female	76	13 (17.1%)	54 (71.1%)	9 (11.8%)
Male	64	7 (10.9%)	49 (76.6%)	8 (12.5%)

### Stress level elements

The analysis revealed varied experiences among the Certified Registered Anesthetists (CRAs): 38.6% felt seldom overwhelmed by work-related stress, while 19.3% occasionally felt incapacitated and overwhelmed. Also, 26.4% never felt fatigued in the morning, and 35% rarely experienced morning fatigue. In terms of feeling drained from work, 17.9% never experienced it, 43.6% rarely did, and 21.4% occasionally felt drained. When it comes to feeling positive or energetic, 25% never felt that way, 32.9% rarely did, and 24.3% occasionally felt positive or energetic. Regarding tolerance to interruptions, 31.4% never found it challenging, while 32.9% rarely did. On the other hand, 22.9% never felt intolerant to hindrances, 40.7% rarely did, 17.9% occasionally did, and 18.6% frequently felt intolerant. Additionally, 23.6% never felt callous toward their patients, while 37.1% rarely did, 22.1% occasionally felt that way, and 17.1% frequently felt indifferent. As for perceiving stress levels among Certified Registered Anesthetists in the Greater Accra region, 17.1% never considered it high, 33.6% rarely did, 36.4% occasionally thought about it, and 12.9% frequently perceived it as high. Finally, 25% never worried about panicking or making mistakes at work, 40% rarely did, 20% occasionally worried, and 15% frequently worried about such situations occurring (Table 2b).

**Table 2b:** Stress level elements.

	Never	Rarely	Occasionally	Frequently	Always
	*n* (%)	*n* (%)	*n* (%)	*n* (%)	*n* (%)
Felt incapacitated or overwhelmed by work related stress?	54 (38.6)	59 (42.1)	27 (19.3)	0 (0)	0 (0)
Feeling tired and fatigued	37 (26.4)	49 (35.0)	35 (25.0)	19 (13.6)	0 (0)
Feel used up/drained from work?	25 (17.9)	61 (43.6)	30 (21.4)	24 (17.1)	0 (0)
Feel positive/energetic.	35 (25.0)	46 (32.9)	34 (24.3)	25 (17.9)	0 (0)
Find it difficult to tolerate interruptions.	44 (31.4)	46 (32.9)	33 (23.6)	17 (12.1)	0 (0)
Intolerant to any hindrances.	32 (22.9)	57 (40.7)	25 (17.9)	26 (18.6)	0 (0)
Feeling of caring less, or becoming indifferent.	33 (23.6)	52 (37.1)	31 (22.1)	24 (17.1)	0 (0)
Panic and making mistakes	35 (25.0)	56 (40.0)	28 (20.0)	21 (15)	0 (0)

### Work-related factors that may affect stress

Table 3 illustrates that 50% of the CRAs encountered negative patient outcomes, such as death or permanent disability, while 50% did not. Moreover, 58.6% of CRAs worked with inadequate or sub-standard resources, while 41.4% did not. Similarly, 58.6% had access to appropriate and qualified theater staff, while 41.4% did not. In cases of difficult tasks, 60% of CRAs worked alone, while 40% did not. Besides, 64.3% of CRAs had the liberty to choose their own methods and techniques, and 35.7% did not. Furthermore, 59.3% had a heavy workload per shift, whereas 40.7% did not have a heavy workload per shift.

**Table 3:** Work-related factors that may affect stress.

Variables and categories	Yes	No
	n (%)	n (%)
Negative patient outcomes at work.	70 (50.0)	70 (50.0)
Inadequate or sub-standard resources, equipment, and supplies?	82 (58.6)	58 (41.4)
Appropriate and qualified theater staff.	82 (58.6)	58 (41.4)
Supervision by a senior colleague at work.	48 (34.3)	92 (65.7)
Working alone on difficult cases.	84 (60.0)	56 (40.0)
Freedom of choosing your own methods and techniques.	90 (64.3)	50 (35.7)
Heavy workload per shift.	83 (59.3)	57 (40.7)
Recognizing for doing a good job.	63 (45.0)	77 (55.0)
Payment of overtime allowance.	40 (28.6)	100 (71.4)
Dissatisfaction with other theater staff.	64 (45.7)	76 (54.3)

### Factors associated with stress-bivariate analysis

#### Association between socio-demographic characteristics and levels of stress

Among female CRAs, 17.1% experienced mild stress, while the majority (71.1%) encountered moderate stress, and a smaller percentage (11.8%) reported severe stress levels (Table 4). For male CRAs, 76.6% reported moderate stress, and 12.5% experienced severe stress levels. However, the association between gender and stress level was not significant (*p* = 0.490). Within the age group of 20–30 years, 77.8% had moderate stress levels; for those between 31 and 40 years, 15.8% had mild stress, 73.7% moderate stress, and 10.5% severe stress. The older age group (>41 years) consisted of 16.2% mild stress, 70.3% moderate stress, and 13.5% severe stress. The relationship between age group and stress level was insignificant (*p* = 0.810). Regarding marital status, single/cohabiting CRAs reported 13.6% mild, 71.6% moderate, and 14.8% severe stress levels, while married CRAs experienced 7.4% mild, 77.8% moderate, and 14.8% severe stress. Among divorced/separated CRAs, 21.9% reported mild, 75.0% moderate, and 3.1% severe stress. The association between marital status and stress level was not significant (*p* = 0.280).

**Table 4:** Association between sociodemographic characteristics and levels of stress.

Variables and categories	Levels of stress				
	Total	Mild	Moderate	Severe	*p*-value
N	140	20	103	17	
Gender					0.490
Female	76	13 (17.1)	54 (71.1)	9 (11.8)	
Male	64	7 (10.9)	49 (76.6)	8 (12.5)	
Age					0.810
20-30 years	27	2 (7.4)	21 (77.8)	4 (14.8)	
31–40	76	12 (15.8)	56 (73.7)	8 (10.5)	
>41	37	6 (16.2)	26 (70.3)	5 (13.5)	
Marital status					0.280
Single/Cohabiting	81	11 (13.6)	58 (71.6)	12 (14.8)	
Married	27	2 (7.4)	21 (77.8)	4 (14.8)	
Divorced/Separated	32	7 (21.9)	24 (75.0)	1 (3.1)	
Number of dependents					0.230
0–2	48	3 (6.3)	39 (81.3)	6 (12.5)	
3–4	78	14 (17.9)	56 (71.8)	8 (10.3)	
>4	14	3 (21.4)	8 (57.1)	3 (21.4)	
Income level (GHC)					0.290
1,500–2,500	23	2 (8.7)	16 (69.6)	5 (21.7)	
2,501–3,500	66	13 (19.7)	48 (72.7)	5 (7.6)	
3,501–4,500	35	4 (11.4)	25 (71.4)	6 (17.1)	
>4,501	16	1 (6.3)	14 (87.5)	1 (6.3)	
Rank/Grade					0.220
CRAs	20	2 (10.0)	13 (65.0)	5 (25.0)	
SCRAs	36	9 (25.0)	24 (66.7)	3 (8.3)	
PCRAs	71	8 (11.3)	55 (77.5)	8 (11.3)	
DCCRAs	13	1 (7.7)	11 (84.6)	1 (7.7)	
Type of health facility					**0.004**
District hospital	49	7 (14.3)	30 (61.2)	12 (24.5)	
37 military hospital	24	5 (20.8)	15 (62.5)	4 (16.7)	
Regional hospital	32	2 (6.3)	30 (93.8)	0 (0.0)	
Teaching hospital	25	6 (24.0)	19 (76.0)	0 (0.0)	
Quasi/Private/Polyclinic	10	0 (0.0)	9 (90.0)	1 (10.0)	
Years of experience (Years)					0.360
0–3	51	10 (19.6)	34 (66.7)	7 (13.7)	
4–7	47	7 (14.9)	37 (78.7)	3 (6.4)	
7–10	19	2 (10.5)	15 (78.9)	2 (10.5)	
>10	23	1 (4.3)	17 (73.9)	5 (21.7)	
Average number of hours spent at work					0.560
<40 h	8	1 (12.5)	5 (62.5)	2 (25.0)	
41-50 h	88	16 (18.2)	63 (71.6)	9 (10.2)	
51-60 h	33	2 (6.1)	27 (81.8)	4 (12.1)	
>61	11	1 (9.1)	8 (72.7)	2 (18.2)	
Number of part-time jobs					0.088
0–1	67	8 (11.9)	49 (73.1)	10 (14.9)	
2–3	59	9 (15.3)	47 (79.7)	3 (5.1)	
>4	14	3 (21.4)	7 (50.0)	4 (28.6)	

The bold values indicates the level of significance of the *p*-values < 0.05.

Among CRAs with 0–2 dependents, 6.3% had mild stress, and 81.3% experienced moderate stress, with 12.5% reporting severe stress. For those with 3–4 dependents, 17.9% had mild stress, 71.8% had moderate stress, and 10.3% had severe stress. For CRAs with more than 4 dependents, 21.4% experienced mild stress, 57.1% had moderate stress, and 21.4% reported severe stress. The relationship between the number of dependents and stress level was not significant (*p* = 0.230). For CRAs with an income between 1,500 and 2,500 GHC, 8.7% experienced mild stress, 69.6% had moderate stress, and 21.7% reported severe stress. Those earning 2,501–3,500 GHC experienced 19.7% mild stress, 72.7% moderate stress, and 7.6% severe stress. For income levels between 3,501 and 4,500 GHC, 11.4% reported mild stress, 71.4% had moderate stress, and 17.1% severe stress. Participants earning more than 4,501 GHC had 6.3% mild stress, 87.5% moderate stress, and 6.3% severe stress. However, the association between stress level and income was not significant (*p* = 0.290). Regarding the rank of the participants, CRAs had 10.0% mild stress, 65.0% moderate stress, and 25.0% severe stress. SCRAs showed 25.0% mild stress, 66.7% moderate stress, and 8.3% severe stress. PCRAs displayed 11.3% mild stress, 77.5% moderate stress, and 11.3% severe stress. DCCRAs exhibited 7.7% mild stress, 84.6% moderate stress, and 7.7% severe stress. The association between stress level and the rank of CRAs was insignificant (*p* = 0.220).

The type of health facilities where CRAs worked also had varying stress levels. For district level facilities, 14.3% had mild stress, 61.2% had moderate stress, and 24.5% had severe stress. At military hospitals, 20.8% reported mild stress, 62.5% had moderate stress, and 16.7% reported severe stress. Regional hospitals showed 6.3% mild stress and 93.8% moderate stress. The teaching hospital revealed 24.0% mild stress and 76.0% moderate stress. Quasi/private/polyclinics showed 0.0% mild stress, 90.0% moderate stress, and 10.0% severe stress. There was a significant association between the stress level and the type of health facilities where CRAs worked (*p* = 0.004).

#### Association between work-related factors and levels of stress

Stress levels were significantly associated with inadequate or substandard equipment, with a *p*-value of 0.014 (Table 5). The table shows that among CRAs working with inadequate or substandard equipment, 8.5% had mild stress levels, 74.4% had moderate stress levels, and (17.1%) had severe stress levels.

**Table 5:** Association between work-related factors and levels of stress.

Variables and categories	Levels of stress				
	Total	Mild	Moderate	High	*p*-value
N	140	20 (14.2)	103 (73.6)	17 (12.1)	
Negative patient outcomes.					0.970
Yes	70	10 (14.3)	51 (72.9)	9 (12.9)	
No		10 (14.3)	52 (74.3)	8 (11.4)	
Inadequate or sub-standard resources, equipment and supplies.					**0.014**
Yes	82	7 (8.5)	61 (74.4)	14 (17.1)	
No	58	13 (22.4)	42 (72.4)	3 (5.2)	
Appropriate and qualified theater staff.					0.645
Yes	82	10 (12.2)	61 (74.4)	11 (13.4)	
No	58	10 (17.2)	42 (72.4)	6 (10.3)	
Supervision by a senior colleague at work.					0.790
Yes	48	8 (16.7)	35 (72.9)	5 (10.4)	
No	92	12 (13.0)	68 (73.9)	12 (13.0)	
Working alone on difficult cases.					**0.040**
Yes	84	12 (14.3)	57 (67.9)	15 (17.9)	
No	56	8 (14.3)	46 (82.1)	2 (3.6)	
Freedom to choose your own methods and techniques					0.525
Yes	90	12 (13.3)	65 (72.2)	13 (14.4)	
No	50	8 (16.0)	38 (76.0)	4 (8.0)	
Heavy workload per shift.					1.000
Yes	83	12 (14.5)	61 (73.5)	10 (12.0)	
No	57	8 (14.0)	42 (73.7)	7 (12.3)	
Recognized for doing a good job.					0.530
Yes	63	7 (11.1)	47 (74.6)	9 (14.3)	
No	77	13 (16.9)	56 (72.7)	8 (10.4)	
Payment of overtime allowance.					0.320
Yes	40	3 (7.5)	31 (77.5)	6 (15.0)	
No	100	17 (17.0)	72 (72.0)	11 (11.0)	
Dissatisfaction with other theater staff.					0.640
Yes	6	11 (17.2)	45 (70.3)	8 (12.5)	
No	76	9 (11.8)	58 (76.3)	9 (11.8)	

The bold values indicates the level of significance of the *p*-values < 0.05.

It was found that (14.3%) of CRAs who encountered negative events were under mild stress levels, 72.3% were under moderate stress levels, and 12.9% were under severe stress. However, this association was not statistically significant (*p* = 0.900). The majority of CRAs who reported working with qualified theater staff were moderately stressed 74.4%, and those who did not report for qualified theater staff also had 72.4% moderate stress. However, working with qualified theater staff was not significantly associated with stress levels (*p* = 0.645).

About 72.9% of participants who were under the supervision of a senior colleague were moderately stressed, while among those who were not under supervision, 73.9% were also moderately stressed. There was no significant association between stress level and supervision by a senior (*p* = 0.790).

Among CRAs working on a difficult case alone, 67.9% were moderately stressed, and 82.1% of those who were not working on difficult cases were also under moderate stress. This association was found to be statistically significant (*p* = 0.040).

The majority of those who had the freedom to choose their methods and techniques had a moderate stress level of 72.2%, and those who were not given the freedom to choose their methods and techniques also had moderate stress of 76.0%. However, no significant association was found between stress levels and having the freedom to choose their methods and techniques.

Among participants with a heavy workload, 73.5% were moderately stressed, while 73.7% who were without a heavy workload had moderate stress. However, this association was not statistically significant (*p* = 1.000).

70.3% of CRAs who were dissatisfied with other theater staff’s attitudes were moderately stressed. 76.3% were not dissatisfied with other theater staff’s attitudes but were also moderately stressed. However, these associations were not statistically significant (*p* = 0.640) (Table 5).

#### Factors associated with stress - ordinal logistic regression

The results from the ordinal logistic regression analysis are summarized in Table 6. The study revealed that only the type of health facility and the use of substandard equipment remained significantly associated with stress in both the unadjusted and adjusted proportional odds ordinal logistic regression models. Working at a teaching hospital, compared to a district hospital, reduced the odds (COR = 0.21; 95% CI = 0.07, 0.65), (AOR = 0.23; 95% CI = 0.07, 0.76) of transitioning from the mild stress category to the moderate/severe stress category. Similarly, participants who reported not working with substandard equipment, as opposed to those using substandard equipment, had lower odds (COR = 0.30; 95% CI = 0.13, 0.69), (AOR = 0.28; 95% CI = 0.11, 0.68) of moving from the mild category to the moderate/severe stress category. Variables such as age, number of hours spent working, dealing with difficult cases, and experiencing a heavy workload were not statistically significant (*p*-value >0.05) in both the unadjusted and adjusted proportional odds ordinal logistic regression models (Table 6).

**Table 6:** Factors associated with stress-ordinal logistic regression.

Effect	Unadjusted model	*p*-value	Adjusted model	*p*-value
	COR [95% CI]		AOR [95% CI]	
Age
20–30 years	1.00 [reference]		1.00 [reference]	
31–40 years	0.58 [0.21, 1.54]	0.274	0.55 [0.19, 1.57]	0.264
>41 years	0.64 [0.21, 1.96]	0.440	0.45 [0.13, 1.57]	0.211
Type of health facility
District hospital	1.00 [reference]		1.00 [reference]	
37 Military hospital	0.48 [0.15, 1.58]	0.228	0.49 [0.14, 1.67]	0.255
Regional hospital	0.46 [0.16, 1.28]	0.137	0.46 [0.15, 1.37]	0.164
Teaching hospital	0.21 [0.07, 0.65]	**0.007**	0.23 [0.07, 0.76]	**0.015**
Quasi/Private/Polyclinic	0.92 [0.20, 4.14]	0.914	1.23 [0.24, 6.34]	0.803
Number of hours spend
<40	1.00 [reference]		1.00 [reference]	
41–50	0.39 [0.07, 2.03]	0.263	0.75 [0.11, 4.78]	0.763
51–60	0.72 [0.13, 4.12]	0.714	1.63 [0.23, 11.78]	0.627
>61–70	0.84 [0.11, 6.51]	0.865	1.06 [0.12, 9.31]	0.959
Substandard equipment
Yes	1.00 [reference]		1.00 [reference]	
No	0.30 [0.13, 0.69]	**0.004**	0.28 [0.11, 0.68]	**0.005**
Working alone on difficult cases
Yes	1.00 [reference]		1.00 [reference]	
No	0.54 [0.25, 1.17]	0.117	0.71 [0.30, 1.70]	0.439
Heavy workload
Yes	1.00 [reference]		1.00 [reference]	
No	1.02 [0.48, 2.19]	0.941	1.44 [0.60, 3.49]	0.414

The bold values indicates the level of significance of the *p*-values < 0.05.

## Discussion of findings

### Socio-demographic characteristics of the CRAs

This study discovered that the majority of Certified Registered Anesthetists (CRAs) experienced moderate stress, aligning with Bakshi et al. findings in the Indian Journal of Anesthesiologist where 99% of anesthesiologists experienced moderate stress (8). Stress is a crucial aspect of human life, and moderate stress, if not effectively managed by the organization, can lead to illness, absenteeism, high turnover, poor performance, employee dissatisfaction, low productivity, and ultimately, poorer client services.

Most of the CRAs in this study were female, aged between 31 and 40 years, and single. This aligns with Morsy & Ebraheem’s findings, which observed that a majority of critical care nurses were female and between the ages of 25 and 30 years. However, most were married (9). However, among the socio-demographic factors studied, only the type of health facility was significantly associated with work-related stress levels. The other sociodemographic factors did not show a significant relationship with stress levels among CRAs.

Anesthesiologists have been found to overwork themselves on certain occasions, leading to mistakes in anesthesia administration due to fatigue (10). The stresses experienced by anesthesiologists arise from difficult job situations, interpersonal conflicts, and career concerns (11). Koshy et al. suggested that long hours of standing, diverse work environments, and travel demands contribute to the high incidence of acid peptic diseases among anesthetists (12). Adzakpah et al. noted that nurses experience stress worldwide due to their close contact with patients, and this stress is often influenced by the type of hospitals they work in and their socio-demographic characteristics (13). Work-related stress has a significant impact on individuals’ health and institutions, causing high turnover rates and absenteeism among workers, ultimately affecting the quality of patient care (14).

### Work-related stress levels among CRAs

The findings reveal that a significant number of Certified Registered Anesthetists (CRAs) experience moderate stress levels. CRAs reported various stress-related elements, such as feeling overwhelmed or incapacitated due to work-related stress, experiencing tiredness and fatigue, feeling used up or drained, experiencing feelings of positivity or energy, finding it challenging to tolerate interruptions, becoming indifferent or feeling less caring toward patients, and worrying about making mistakes. These responses are expected because stress sometimes prompts individuals to display abnormal behaviors due to the release of cortisol in the brain. This study’s findings are consistent with Bakshi et al.’s research in 2017, where 69% of anesthesiologists rated their stress levels as moderate, with 22% experiencing extreme stress and 9% reporting minimal stress. Respondents in that study also exhibited signs of stress, such as morning tiredness a decreased level of concern and empathy toward patients. There was a significant association between the stress symptoms and the burnout reported by the participants (8).

Moreover, the current study’s results are in line with Adzakpah et al. (13) study among nurses in hospital settings, indicating that work-related stress among Certified Registered Anesthetists is a significant concern in healthcare environments. Embriaco et al. noted that working in intensive care units and anesthesia departments is more stressful compared to other departments (15). Procedures such as intubation and anesthesia inductions contribute to heightened stress levels, often resulting in symptoms like palpitations, tachycardia, and other circulatory issues. Additionally, studies have shown that on-call doctor anesthesiologists are considered one of the most stressed groups, experiencing high levels of stress within their roles (12).

### Factors associated with stress

The study revealed that stress among Certified Registered Anesthetists (CRAs) was not significantly associated with gender, age, marital status, rank, income, number of dependents, number of part-time jobs, or various work factors. However, a significant association was observed between stress and the type of health facility. The correlation between stress and the type of health facility might be attributed to the nature of referral centers or facilities situated in densely populated areas. Referral centers, such as the Korle-Bu Teaching Hospital, encounter higher patient volumes, leading to elevated stress levels among CRAs. Research by Bhutani et al. supported the notion that physicians in private organizations experienced higher job satisfaction compared to those in government facilities, as government hospitals often lacked proper resources and equipment, resulting in poor working conditions (16).

The study also identified a significant association between work-related stress and the absence of adequate or standard equipment. Inadequate resources can significantly impact the workload and the level of stress experienced by CRAs. Factors such as working alone on difficult cases, heavy workload per shift, negative patient outcomes, appropriate theater staff, supervision by senior colleagues, recognition for good work, freedom to choose one’s techniques, overtime payments, and dissatisfaction with other theater staff were not significantly related to stress in the logistic regression. Although these factors were not directly associated with stress, they could contribute to other health issues or cause a lack of motivation to achieve optimal results.

Kokoroko and Sanda’s research in 2019 on nurses at Komfo Anokye Teaching Hospital (KATH) highlighted that workload was a major contributor to stress among nurses, leading to dissatisfaction and a lack of motivation for high-quality performance at work (17). In another study, Jenkins & Wong found that factors like time constraints, interferences with home life, medico-legal concerns, and clinical problems significantly contributed to stress (18). The absence of significant associations in some factors might not directly relate to stress but could affect other aspects of the professionals’ health and work motivation.

### Study limitation

While this study has yielded important findings, we acknowledge some limitations. Foremost, the results obtained from this research cannot be broadly applied to all Certified Registered Anesthetists (CRAs) across Ghana, as the study was exclusively conducted within the Greater Accra Region. Therefore, any interpretation of the results should be done with caution as the results may not be generalizable. Additionally, access to participants was hindered by the challenging circumstances posed by the COVID-19 pandemic, as potential respondents declined to participate in the study because they were not comfortable with the electronic medium the study adopted for data collection. Notwithstanding, the census approach ensured that as many respondents as possible were recruited which ensured good level of rigor.

## Conclusion

The study findings indicated that most Certified Registered Anesthetists (CRAs) in the Greater Accra Region experienced moderate stress levels. The ordinal logistic regression model revealed that only the type of health facilities and working with substandard equipment significantly influenced stress levels. In contrast, factors like working alone on difficult cases, heavy workload, and certain sociodemographic characteristics (gender, age, rank, income, number of dependents, and part-time jobs) were not significant. Moreover, the study highlighted the various coping strategies adopted by CRAs to deal with work-related stress, including denial of guilt, substitute gratification, situation control, reaction control, positive self-instructions, escape, peer support/spending time with family and friends, and resignation. Establishing well-structured working environments, motivating CRAs, offering standardized resources, and facilitating access to essential information were highlighted as critical. These changes were expected to enhance the psychological readiness of CRAs and improve their autonomy, confidence, and strength, ultimately leading to increased job satisfaction, a sense of personal accomplishment, and reduced work-related stress. Based on the moderate level of work-related stress observed among CRAs, the study suggests that the Ministry of Health, in collaboration with various health service organizations, should conduct educational programs and counseling for CRAs to create awareness about the demands of their job and provide psychological preparedness.

The study recommends the implementation of tailored stress management initiatives for Certified Registered Anesthetists (CRAs) within healthcare organizations, emphasizing the unique stressors faced by female and younger CRAs. Utilize strategies like workload distribution and resource allocation to address facility-specific stress factors, enhance working conditions for sustained positive impacts on CRAs’ well-being and job satisfaction.

## Data availability statement

The original contributions presented in the study are included in the article/supplementary material, further inquiries can be directed to the corresponding author.

## Ethics statement

The studies involving humans were approved by Ghana Health Services Ethics Review Commitee. The studies were conducted in accordance with the local legislation and institutional requirements. The participants provided their written informed consent to participate in this study.

## Author contributions

DS: Conceptualization, Data curation, Formal analysis, Investigation, Resources, Writing – original draft, Writing – review & editing, Funding acquisition, Methodology. AS: Data curation, Funding acquisition, Investigation, Methodology, Writing – original draft, Project administration. RO: Data curation, Funding acquisition, Investigation, Writing – original draft, Conceptualization, Formal analysis, Resources, Supervision, Validation, Writing – review & editing. SB: Conceptualization, Data curation, Formal analysis, Investigation, Resources, Supervision, Validation, Writing – original draft, Writing – review & editing, Visualization.
